# Integrated knowledge translation in nursing homes: exploring the experiences of practice development nurses

**DOI:** 10.1186/s12913-021-07282-7

**Published:** 2021-11-29

**Authors:** Trine-Lise Dræge Steinskog, Oscar Tranvåg, Donna Ciliska, Monica Wammen Nortvedt, Birgitte Graverholt

**Affiliations:** 1 Western Norway University of Applied Sciences, P.O. Box 7030, N-5020 Bergen, Norway; 2 Norwegian Research Centre for Women’s Health, Oslo University Hospital, Rikshospitalet, P.O. Box 4950 Nydalen, 0424 Oslo, Norway; 3 McMaster University, 1280 Main St W, Hamilton, ON L8S 4L8 Canada

**Keywords:** Nursing homes, Integrated knowledge translation, Evidence-based practice, Quality improvement, Practice development

## Abstract

**Background:**

Practice Development Nurses (PDNs) in Norwegian nursing homes (NHs) hold a specific responsibility for knowledge translation in this increasingly complex healthcare setting. They were involved as end users in an integrated knowledge translation (IKT) study, developing, testing and evaluating the IMPAKT (IMPlementation of Action to Knowledge Translation) intervention. PDNs participated in an educational programme tailored to their own defined needs. In a second intervention component, the PDNs applied their new skills with facilitation, in implementing the National Early Warning Score (NEWS2) in their respective NHs. The aim of this study was to explore 1) the PDNs’ experiences of participating in an IKT educational intervention, and 2) how they applied the learning in planning, tailoring and initial implementation of the NEWS2.

**Methods:**

This is a qualitative exploratory study based on a phenomenological hermeneutical method. Study participants were PDNs working in the nine NHs in the intervention group of the IMPAKT trial. We conducted nine in-depth interviews and eight non-participatory observational sessions of the intervention delivery.

**Results:**

The PDNs expressed that the educational programme met their needs and enhanced their understanding about leading knowledge translation (KT). They reported a move from operating in a “big black box of implementation” to a professional and structured mode of KT. The gamechanger was a shift from KT as the PDNs’ individual responsibility to KT as an organizational matter. The PDNs reported enhanced competencies in KT and in their ability to involve and collaborate with others in their facility. Organizational contextual factors challenged their KT efforts and implementation of the NEWS2.

**Conclusions:**

This study demonstrates that an IKT approach has the potential to advance and improve staff competencies and NH readiness for KT. However, individual motivations and competencies were challenged within an organizational culture which was less receptive to this new leadership role and level of KT activity.

**Supplementary Information:**

The online version contains supplementary material available at 10.1186/s12913-021-07282-7.

## Background

Worldwide, we see populations aging, with increasingly complex care needs that require skilled, high-quality nursing home (NH) care [1–3]. Internationally, evidence-based practice (EBP) is linked to improved quality of care and healthcare outcomes [4, 5], and NHs are increasingly challenged to implement EBP in their organizations due to knowledge-to-action gaps [6, 7]. These gaps persist, partly due to a knowledge production problem, where research fails to address priority questions of knowledge users. But more often, healthcare organizations fail to translate knowledge effectively into practice [8]. An adequately trained workforce is vital to the provision of efficient and effective care in NHs globally [9, 10]. In Norway, a discrepancy has been identified between expected and actual competence among nurses working in NHs, implying that further development of knowledge and skills in this setting is warranted [11].

Knowledge translation (KT) is an umbrella term for moving knowledge into healthcare practice and policy. KT includes the synthesis, dissemination, exchange and application of knowledge to improve health and strengthen the healthcare system [12]. Traditional models of knowledge creation and transfer are increasingly being replaced by inclusive research approaches, which integrate the complexities of healthcare environments with relevant needs of knowledge users [6]. Integrated knowledge translation (IKT) is positioned within this paradigm of inclusive research, to address the challenge of under-utilization of research [13]. Central to the IKT process is the involvement of knowledge users as partners throughout the research process [12]. One potential benefit of IKT is mutual capacity development in the partnership [14, 15] This is thought to create trust in a shared vision that can further increase capacity for KT [16].

Existing evidence relating to IKT indicates that the involvement of stakeholders in a participatory research approach may strengthen the relevance of research evidence created and facilitate its uptake in practice [17, 18]. But literature on such partnerships in NH settings is scarce [19–21]. Despite a rapidly growing body of implementation science in healthcare, we still know little about context-specific implementation in NHs [22]. Research with an IKT approach in the long-term care settings is warranted to understand relevant challenges and how these can be met [19, 23, 24]. By involving relevant knowledge users, solutions can be identified that reflect the realities of the setting, thereby heightening the potential for success when they are implemented [25–27].

### The IMPAKT study

The IMPAKT (IMPlementation of Action to Knowledge Translation) trial (ISRCTN Trial ID: 12437773) addressed the knowledge-to-action gap from the perspective of healthcare professionals in NHs. Using an IKT approach, the intervention was developed in close cooperation with a publicly funded NH organization. To develop the intervention, interviews with PDNs and observations in NHs were undertaken to gain an understanding of context-specific KT challenges and educational needs [28].

The IMPAKT intervention consisted of two key components. The first was an educational programme addressing identified KT learning needs, including basics of EBP, such as finding and appraising research, and preparing the participants to write an Action plan for the implementation of NEWS2. An outline of the educational component of the IMPAKT intervention is presented in an additional file [additional file 1.]. The programme centred around the Knowledge-To-Action (KTA) framework [29], which was developed to guide the implementation of evidence-based interventions. It outlines a process representing the activities involved in KT, including adaptation of knowledge to local context, assessment of barriers and facilitators to its use, in a tailored strategy for implementation [30]. During the programme, participants from each intervention NH collaborated to develop Action plans for implementing the NEWS2. The decision to implement the NEWS2 was made in consultation with PDNs, then physicians and senior managers of the NH organization were involved as stakeholders [31]. The National Early Warning Score-2 (NEWS2) is a systematic scoring tool used to detect deterioration in patients [32].

The second intervention component was a facilitation-upon-implementation phase, during which PDNs in the nine intervention NHs applied their learning and executed their Action plans. The Action plan template followed the phases of the KTA framework and included planning, implementation and evaluation. Planning involved identifying stakeholders relevant to implementation of the NEWS2, followed by ascertaining facilitators and barriers to its use, and specific implementation measures [29]. PDNs led the process in their respective NHs, facilitated by researchers. Further details about the IMPAKT trial are described elsewhere [33, 34].

The aim of this study was to explore 1) the PDNs’ experiences of participating in an IKT educational intervention, and 2) how they applied the learning to their planning and tailoring during the initial phase of implementing the NEWS2.

## Methods

This study was based on a qualitative, exploratory design, utilizing in-depth interviews [35] and non-participant observations [36, 37] to collect data. Data were analysed using Lindseth and Nordberg’s [38] phenomenological hermeneutical method for researching lived experience. The method is inspired by Ricoeur’s [39] theory aimed at interpreting and understanding the meanings of a phenomenon, in this case, the PDNs’ experiences of working with KT. Interventions within healthcare need to be understood in ways that are responsive to the complexities of programmes, people and places [40]. As such, Ricoeur’s considerations of the relationship between phenomenology and hermeneutics as a relationship of reciprocity [39] have the potential to contribute to a deep, interpretive understanding of PDNs’ experiences. Whereas phenomenology focuses on the lived experience of humans, hermeneutics uses lived experience as a tool for gaining an understanding of the social, cultural, political and historical context in which those experiences occur [41, 42]. Our reporting of the study’s findings is in accordance with the Standard Recommendation for Qualitative Research (SRQR) [43].

### Setting and participants

The study took place in an urban-suburban municipality in western Norway. The municipalities in Norway are, by law, responsible for NH services [44] and are required to improve healthcare quality and safety [45]. Nine of the 23 public NHs in the municipality were randomized to participate in the IMPAKT intervention group [33]. Public NH facilities in Norway are similar in terms of funding, commitments and affiliation to the municipal NH organization and admission of new residents [2]. Every NH in this organization has one Practice Development Nurse (PDN), who holds a key responsibility for quality improvement and professional development of staff across the institution. The formal educational requirement of the PDN is a bachelor’s degree in nursing, but most PDNs have post graduate education (Table 1). PDNs work closely with the management and all wards in the NH, and report to the NH Director. Their duties and responsibilities vary between the NHs [3]. In our IKT approach, the PDNs were involved as end users in the process of developing, testing, and evaluating the IMPAKT intervention.

**Table 1:** Characteristics of the PDNs

Age (n)	< 40 years (3)	40–50 years (4)	> 50 years (2)
Years in the position as PDN (n).	< 1 (1)	1–5 (6)	> 5 (2)
Percentage of full-time equivalent as a PDN (n)	20% (2)	40–80% (3)	100% (4)
Postgraduate education (n)	None (2)	Clinical certificate^a^ (6)	Master’s level (1)

^a^Courses between bachelor and master’s level within the fields of oncology, health environment and safety, project management, quality improvement supervisor training, pedagogy, palliative care, geriatrics and paediatrics

The nine PDNs employed in the intervention NHs (Table 1) were recruited by phone to participate in in-depth interviews. Arrangements to observe intervention delivery were scheduled with the PDNs via email and phone.

### Data collection and tools

**Fig. 1:**
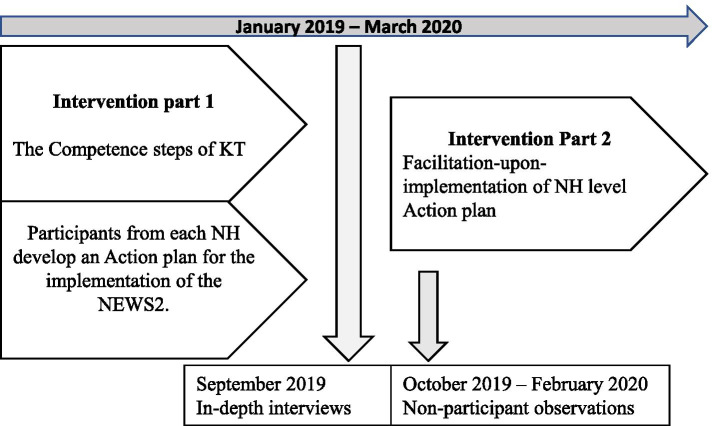
Outline of the IMPAKT intervention and data collection times

We carried out nine in-depth interviews during September 2019. Eight non-participant observational sessions of the NEWS2 implementation took place between October 2019 and February 2020 (Fig. 1).

The interview guide [see Additional file 2] was shared with the PDNs ahead of the interviews to encourage reflection. The interview questions were open and exploratory. They focused on the PDNs’ experience of participating in the KT educational programme, and how they transformed their learning into developing the Action plan and initial implementation of the NEWS2. The interviews took place in the NHs and lasted 60 min, on average.

Non-participant observations were performed to collect data on how the PDNs chose to tailor the initial phase of implementing the NEWS2. Typically, these activities were dissemination and educational sessions, varying in length and form, from brief 30-min lunchtime sessions to a full day. The first author made field notes focusing on delivery of the sessions in terms of numbers of participants, their occupation and response, time and setting, as well as tools utilized, activities performed, and materials distributed. After each observation session the field notes were read and reflected upon, writing longer sentences where the observations notes needed more substance, as well as formulating an additional summary ensuring that all observations were documented.

### Data analysis

The interviews were transcribed verbatim, and along with the field notes, the empirical data were analysed and interpreted using a phenomenological hermeneutic approach [38]. Recording discourses in writing creates a distance that releases the meaning from the told narratives, whereby an interpretation option opens [39]. By utilizing this approach, we were able to move beyond a surface interpretation to an in-depth interpretation of the transcribed empirical material [39]. We employed a three-step interpretation, including a *naïve reading*, *structural analysis* and *comprehensive understanding* in order to develop an understanding of the PDNs’ perceptions [38]. Firstly, a *naïve reading* was conducted to grasp an overall impression of the text. This step entailed reading the text with an open, inductive mind. This allowed access to the PDNs’ beliefs about the educational programme and uncertainties about implementation. When moving to the second step, the *structural analysis*, we identified meaningful units of the text, before condensing them and formulating sub-themes and themes in relation to the research question and the context of the study (Table 2).

**Table 2:** Example of the analysis process

Meaning Unit	Condensation	Sub-theme	Theme
*“The Action plan template provides us with a recipe for which areas we need to pay attention to, to succeed with the implementation. It made me aware of different steps we should consider and investigate”- PDN 1.*	The PDN valued (the introduction of) tools that gave a sense of direction in the implementation process.	KT tools added structure to planning the implementation.	The need for undertaking a systematic process for planning KT.

During the structural analysis, we identified patterns of meaningful consistency, which enabled us to understand and describe the substance of the texts. Finally, we developed a *comprehensive understanding* of the texts as an interpreted whole. Our aim was to obtain a critical in-depth understanding, with members of the research team reflecting upon and discussing their initial naïve understandings and subsequent findings from the structural analysis [38, 39]. Quotes were selected that best illustrated emerging themes and, which best substantiated the findings.

## Results

We identified three themes and six sub-themes expressing the PDNs’ experiences of the KT educational programme and applying their learning to planning, tailoring and initial implementation of the NEWS2 in the NH setting (Table 3).

**Table 3:** Identified themes and subthemes

Themes	Expanding understanding of the KT role	The need for undertaking a systematic process for planning KT	Confronting the realities of organizational structures when implementing KT
Sub-themes	Enhancing professionalism through gaining competence in KT strategies	KT tools added structure to planning the implementation	Experiencing unpredictability in the organization
	Broadening the perspective of KT responsibilities via shared learning	Confirming the need for leadership involvement in KT	Acknowledging the need to shift from passive to active translation

### Expanding understanding of the KT role

Among the PDNs, a true systematic approach to implementation was not common practice prior to participating in the intervention. Their partaking in the KT educational programme made them recognize that implementation, as defined in the programme, was a core responsibility of theirs. Formerly, when introducing new routines to their NH, they mostly applied a standard teaching and printed material distribution strategy, regardless of the implementation issue they worked on. They realized that this led to haphazard results, as local factors and topic-specific issues were overlooked. The PDNs felt that they had previously lacked the tools to deal with the complexity of introducing new knowledge to a hectic healthcare setting. They valued how the programme helped them to unwrap the “black box” of implementation, providing them with a system of concepts and tools that expanded their understanding of implementation mechanisms, and the complexities and challenges of implementing the NEWS2 or any other implementation.

*“The new level of understanding implementation processes was the most useful to me. There are so many elements to implementation that I never considered; capacity … capacity building, motivation, facilitation, evaluation – it’s not exactly straightforward”- PDN 3.*

There seemed to be unanimity among the PDNs in terms of how the programme made them realize their deficiencies in implementation capacity. They all commented on how introduction of the KTA model was helpful for breaking down the implementation process, with the potential to clarify and organize their responsibilities.

#### Enhancing professionalism through gaining competence in KT strategies

All PDNs stated that the KT educational programme suited their needs. Several valued the structure of the programme, which started with a basic introduction to EBP and KT. Some underlined how relevant clinical examples, and the support of relevant research evidence in presentations and the curriculum, made the content credible and easy to comprehend. One PDN exemplified how a session on knowledge-to-action gaps made her question her practice.

*“The programme provided me with new perspectives to the discussion of how we justify our practice in NHs. It motivated me to consider our own knowledge-to-action gaps and how we can reduce them” – PDN 2.*

Several shared that their search for evidence was more efficient using the PICO format for questions and filtering for more highly synthesized evidence. For instance, some applied their new tools to formulate answerable questions in a PICO format. The majority expressed an increasing reliance on the Norwegian Electronic Health Library.

*“Clinical questions arise all the time, but we were used to finding solutions within our organization. Now I think differently [;] we should approach questions more systematically and search for answers in research” – PDN 8.*

The PDNs seemed to be united in having a greater sense of the value of research evidence as support for decisions. In turn, this enhanced their professionalism. Still, reading and appraising research evidence remained an explicit barrier once the educational programme was over. Several questioned whether retrieving and appraising evidence, as opposed to facilitating implementation, should be at the core of their role. It was evident that most preferred to be involved in the latter.

#### Broadening the perspective of KT responsibilities via shared learning

Organized and informal conversations and debates enhanced PDNs’ insight into alternative approaches to KT responsibilities. This pedagogical approach was appreciated, and several made reference to these opportunities for sharing among them.

*“The conversations with the other PDNs were perhaps the most valuable element of the programme. We were able to talk about the EBP and KT and gain insight into how other PDNs deal with implementation”- PDN 7.*

Most PDNs were motivated by the idea of a common goal in terms of implementing the NEWS2 across different facilities. They believed that such an alliance of PDNs contributed to the implementation process. Some contacted their peers for advice on tailoring implementation of the NEWS2. Others exchanged educational materials prepared for the purposes of introducing the NEWS2 to NH staff.

### The need for undertaking a systematic process for planning KT

Even if the PDNs were unfamiliar with systematic approaches to implementation, the content of KT felt recognizable and relevant. Unused to preparing strategies in writing, they found the Action plan template useful as a guide to planning implementation of the NEWS2. Overall, they found themselves more organized and ascribed this to the educational programme’s tools and concepts.

*“The educational programme made me more conscious about how I think we should work with quality improvement efforts and implementation in the nursing home. Even if the academic wrapping was new, it fits well within my job as a PDN and my areas of responsibility” – PDN3.*

The PDNs felt that the programme helped them to plan the NEWS2 implementation in a more stepwise, systematic manner.

#### KT tools adding structure to planning the implementation

Some described how the Action plan contributed to a less personal and more organizational responsibility. By systematically identifying local stakeholders, barriers and facilitators, implementation of the NEWS2 became a collective effort.

*“Previously, if someone left their position, the work followed the person “out the door”. With the Action plan I believe that even if I leave, our work is documented. We developed the plan as a team, and it can be fulfilled by others. It simply makes implementation less person-dependent” – PDN 2.*

The Action plan helped the PDNs map local factors that might affect implementation of the NEWS2. They described how they had identified facility-specific knowledge-to-action gaps in relation to the NEWS2. Moreover, they identified stakeholders, barriers and facilitators, and could then adapt and tailor the NEWS2 implementation to their specific context and the NH staff. They embraced the Action plan template as a means of helping them to organize their assessments, and several underlined that the Action plan could serve as a reminder.

#### Confirming the need for leadership involvement in KT

The educational programme reinforced the PDNs’ conviction that active leadership support was needed in implementation projects. They became more insistent on leadership involvement during planning and implementation of the NEWS2. One of the PDNs expressed it like this:

*“My leaders were involved from the very beginning, first by identifying key staff in their wards [for participation in the educational programme]. The leaders are well informed of the whole process, about the potential benefits and how we can take action to achieve them. I’m dependent on the staff to participate in the implementation of NEWS2 and leaders’ support and follow-up. If they hadn’t been involved, this would have been very difficult” – PDN 6.*

The majority experienced a positive attitude among the NH management towards implementation of the NEWS2. The PDNs believed that prioritization by and the attendance of both researchers and the municipal agency provided an organizational commitment to the implementation process. While most directors of NHs naturally became involved, others did not. To compensate, some invested extensively in encouraging leader involvement. A few NH directors participated in the educational programme. PDNs who were accompanied by their leaders valued their presence, expressing that this strengthened the prospects of KT efforts.

### Confronting the realities of organizational structures when implementing KT

All PDNs worked to tailor a KT Action plan for implementing the NEWS2 in their NHs. Yet they painted a complex picture of critical conditions affecting the implementation process.

#### Experiencing unpredictability in the organization

Concurrent interferences arose in most of the NHs and competed with the implementation project. Disruptions included ongoing reorganization of the NH organization, staffing instability and tight budgets. Unpredictability in staffing was viewed as a critical barrier to implementation of the NEWS2. The benefit brought about by having participated in the educational programme along with colleagues in their facility partly disintegrated when staff left their jobs.

*“Staff turnover is a challenge and affects the progress of implementing NEWS2. Even if I’m devoted, the investment in staff is of no use if they leave. If we could only keep them” – PDN 8.*

In addition, several NHs experienced turnover among physicians and top management. PDNs generally acknowledged the importance of involving physicians early on. Physician involvement ranged from “drivers of change” to non-involvement. Another general challenge was reaching out with information about the NEWS2 to staff, not least to the non-permanent staff and those who work nights and evenings. Tight budgets jeopardized sick-leave replacements, which, in turn, negatively influenced the implementation project.

*“In my NH, the subject of conversation is economy and sick leave. When shifts are not covered, the remaining staff obviously can’t leave for professional development. This permeates the entire NH” – PDN 9.*

Cost containment and daily operations increasingly put restraints on KT possibilities for most PDNs and general staff. This had various expressions when PDNs were observed conducting the NEWS2 training. In some NHs, PDNs were unable to stick to their original Action plan and instead organized ad hoc activities. These unplanned sessions had several disturbances, including alarms and staff being called upon by the wards. In one session, staff appeared rather restless and expressed discomfort at leaving the hectic ward. In response, the PDN promised to make the session short and consequently had to rush through her session on the NEWS2, leaving out certain parts. In contrast, some PDNs offered full-day sessions with practical rehearsals. In these cases, cover was arranged on the wards for the staff involved, so they were able to focus in an undisturbed environment. The shorter sessions focused on a minimum presentation of the NEWS2 tool, with limited dialogic learning. Full day sessions often included reflective learning, including simulation training, videos and practicing the clinical procedures and observations included in the NEWS2.

Despite the positivity and motivation shown towards implementation of the NEWS2, some PDNs worried that unpredictability could affect their Action plans. Several found it challenging to settle actual dates for educational activities. Others emphasized the importance of close collaboration with their leaders in order to work around this, towards their common goal of implementing the NEWS2.

*“I need a leader with a clear strategy, then I will know what she expects from me” – PDN 4.*

Leadership involvement determined how systematically the PDNs could disseminate information about the NEWS2. In some NHs, the director attended the training sessions and expressed support for the implementation. Notes from observations of these sessions revealed an engaged group. Despite formal support from NH management, some PDNs felt that this did not extend beyond the initial agreement, and the PDNs had to work after hours to keep up with their Action plan.

Interviewees hoped to integrate professional development into the long-term strategies of NHs. This would legitimize prioritization and support when other requirements distracted them from focusing upon implementation projects. Earlier, they had uncritically accepted unrealistic KT expectations in their organization, whereas now, several felt that they needed more organizational support to be able to prioritize the NEWS2. This awareness reflected the need to create a systematic KT strategy. The PDNs expressed that the educational programme had triggered this awareness.

#### Acknowledging the need to shift from passive to active translation

PDNs recognized the need for a shift in terms of how they lead and facilitate KT, from passive to more active implementation, with hands-on training if needed. Moreover, it was clear that KT required more tailored facilitation, with follow-up, in order to address questions and uncertainties. Not least, they acknowledged the need to address underlying attitudes crucial to the success of KT interventions and tailor these accordingly. In this way, the PDNs moved from subtle notions of how attitudes permeate the way in which wards work in relation to implementation, to systematic and actionable observations.

*“In one of our wards, the ward nurse is goal-oriented with clear expectations [of] the staff. She is a driving force in the implementation of NEWS2. The staff appreciate this and want to perform well” – PDN 8.*

Several pointed to the Action plan and how it added value in the implementation process. The identification and involvement of stakeholders specific to the NEWS2 represented a new way of involving the workplace. By identifying stakeholders specific to the NEWS2, they planned to involve them more closely than they had before. When PDNs had worked with the leadership on the Action plan, they felt supported during the training sessions. PDNs experienced less resistance towards implementation of the NEWS2 than in previous implementation projects. All of them reflected upon how inclusion of care staff in a democratic decision to implement the NEWS2 had left PDNs feeling optimistic about the KT project’s relevance and usefulness. This optimistic tone was apparent during the observations, where staff expressed that the NEWS2 was a tool which they had previously lacked and that they looked forward to applying it to patients. Nevertheless, the PDNs planned continuous, active facilitation of the implementation to remind and support the staff. There was a shift in the PDNs’ KT strategies – from helping changes happen to making them happen.

## Discussion

The themes identified from the data – 1) *expanding understanding of the KT role;* 2) *the need to undertake a systematic process for planning KT;* and 3) *confronting the realities of organizational structures when implementing KT* – describe the PDNs’ experiences of participating in an IKT educational intervention. Firstly, the PDNs unanimously agreed that the programme was relevant to their responsibility to lead KT in their NHs. Secondly, they reported having moved from operating in the “big black box of implementation” to a professional and structured mode of KT. When it came to applying what they learned, the most significant gamechanger was the move from KT as their individual responsibility to KT as an organizational matter. Thirdly, despite these positive findings, the participants vividly recounted how the organizational contextual conditions made it challenging for them to implement the NEWS2.

Within the study’s IKT approach, the PDN role was identified as pivotal to KT in nursing homes by leaders in the NH organization [28]. Decision-makers in the organization appointed participants for the educational programme, which, in turn, seemed to impact on the programme’s importance and relevance for the NH [34]. We identified some of the same advantages as IKT projects reported in the literature, such as increased learning [46] and empowerment to achieve change [47]. These gains may enhance professional development by triggering the participants’ investment in implementation efforts [4]. Professionalism, in the sense of possessing the competence to understand and use research findings, is also described as a facilitator of implementation [48] and a core competency of KT [49]. Our findings suggest that the PDNs experienced relevant learning outcomes, and readiness to lead and tailor implementation of the NEWS2. This broadened confidence and understanding triggered their motivation to work with KT processes in general and, specifically, to implement the NEWS2.

Understanding and preparing the organizational culture for change has previously proven critical to implementation efforts [49, 50]. While developing their Action plans to implement the NEWS2, PDNs experienced the rewards of collaborative efforts involving stakeholders. Haycock-Stuart and Kean [51] argue for the advantages of collaboration between management and frontline nurses, as a shared vision leads to more successful integration of policies into practice [51]. Jull et al. [52] further contend that the IKT approach enables participants to advance their understanding of organizational processes through cooperative practices. The shift in focus from individual to an organizational level was highly appreciated and desired by the PDNs. Due to previous experiences where implementation processes had seemed to be personal battles, this collective effort eased their burden of responsibility. They felt that the Action plan template helped them to work stepwise with other stakeholders in the organization. Together, they negotiated a KT plan specific to implementation of the NEWS2 in their facility. The PDNs’ efforts to collaborate within and across the NH facilities were emphasized as being essential in order to facilitate a supportive culture of KT and readiness to implement the NEWS2.

In their development of Action plans for the NEWS2, PDNs mapped potential barriers. As implementation of the NEWS2 proceeded, new and different challenges became apparent. Research reveals that barriers to KT are often related to contextual conditions beyond the individual’s control [53, 54]. Organizational barriers to implementing new knowledge are comprehensively documented across healthcare organizations and include low organizational recognition, lack of leadership involvement, high workloads, limited resources and time, and resistance to change [46, 55, 56]. Everyday NH practice is characterized as fluctuating [57], with a “culture of busyness” [54] that values efficiency [58]. This is similar to our findings, where PDNs expressed unpredictable workdays with conflicting priorities and demands. Ree et al. [59] found similar results in another Norwegian context. However, they argue that managers in primary care play an important role in negotiating the dynamic contextual environment. The ability to prioritize quality work requires specialized competence and the possibility to delegate responsibility [59]. We found considerable variation in how PDNs created opportunities for initial dissemination activities of the NEWS2. This disparity reflected their level of authority and autonomy, their role and relationship with the NH management and organization, and existing frames and the culture governing educational activities within the NH. In an earlier study we found the PDNs played a key role in driving quality work in NHs [3]. The PDNs expressed conflicting expectations, accumulation of tasks, lack of predictability and leadership commitment as factors threatening their opportunity to fulfill their role [3]. After their participation in the educational component the PDNs requested more active leadership support beyond verbal assent to confront contextual conditions that challenged their KT effort to implement NEWS2.

### Implications

Implications for practice and managers: This study suggests that IKT is a promising, valued and relevant approach to quality improvement work in NHs. We find that its main potential lies in the systematic approach and involvement of the entire organization. But the integration of KT into NHs requires specific commitment and competence beyond what is perhaps present in the workforce, including leaders, today. Lack of KT awareness among NH managers directly influence how staff perceive usefulness and relevance of KT. Creating a KT culture within an organization requires capacity, resources, and a culture for continuous learning. We found that IKT has a potential to bridge these premises by addressing the need for structures, like clear leadership roles to support EBP and KT. Implications for policy makers: Organizational commitment is of the utmost importance when it comes to supporting and promoting ongoing professional development facilitating KT. This should go hand in hand with long-term plans for quality work, which establish clear KT expectations, including competence levels, roles and responsibilities. In turn, this may foster dialogue and action promoting a KT culture, and it may be a way to integrate existing structures that support KT and help get rid of the hindrances.

Implications for education: Our results indicate that KT competencies were largely new to our study sample and were apparently lacking in terms of formal competence. Based on this, we question how well educational health programmes are harmonized to prepare candidates for KT work in Norwegian healthcare today.

Implication for research: In further studies of KT in nursing homes, we would support the use of Gagliardi and colleagues’ conceptual framework for supporting and optimizing KT [46]. This framework suggests making use of the strategies raised by PDNs in our study, and its application is likely to enhance future IKT initiatives.

### Strengths and limitations

A strength of this study was the comprehensive unfolding of PDNs’ lived experience through the phenomenological hermeneutical methodology. Inspired by Ricoeur [39], Lindseth and Norberg [38] argue that the goal of encouraging participants to reflect and share their experiences is to make them discover and articulate their perceptions of the phenomenon under investigation. Lillehagen and Vøllestad [60] contend that KT refers to a form of activity that may be difficult to articulate: “We know that we are doing it, but we do not know what we do when we are doing it.” (p. 3). In our study, we therefore paid attention to both explicit and tacit knowledge by utilizing a combination of interviews and observations. Tacit knowledge has important implications for nurses’ decision-making and KT strategies but has previously not gained much attention [61]. Another strength was the timing of the interviews; they were conducted between the educational and facilitation component, thus providing substantial here-and-now perspectives.

This qualitative encounter has helped us identify local differences across the intervention NHs that may strengthen the value of the future process evaluation of the IMPAKT trial. Our study holds the risks of social desirability and reporting bias. There is a chance that the PDNs may have associated the researcher (first author) with the IKT partnership. The researcher consciously avoided active participation and attempted to maintain clarity in terms of her role at all times, for instance, during observations of the educational programme and local implementation strategies in the NHs. There is a risk that the PDNs withheld negatively loaded information out of loyalty to the organization. Our impression is, however, that the PDNs were not inhibited by this and were, in fact, open and willing to convey shortcomings and constructive feedback.

The trustworthiness of this study has been sought through emphasizing the transparency of the research process. We endeavoured to maximize credibility by performing investigator triangulation [62], with three researchers reading the interviews and observation notes, followed by a reflective analytical process. Dependability and confirmability were sought by an audit trail of the data collection [62], containing summaries and reflections of data, and subject to discussions in our research team. We collected sufficient data to increase transferability by providing a thorough description of the PDNs’ experiences, illustrated by several direct quotes and observations, included in this article. IKT is a way of doing research that theoretically may increase the chances of the results being applicable to the population under study [63]. As such, we placed an emphasis on writing the findings in language that reflected participants’ expressions as closely as possible [41, 62]. Finally, according to Ricoeur [39], a study’s reliability lies in its recognition of others. Against this backdrop, we argue that our findings are relevant to implementation in healthcare, particularly in NHs.

## Conclusion

To the best of our knowledge, this paper is the first to report experiences from an IKT educational intervention in a NH setting. KT capacity emerged as a prerequisite to roles in healthcare that hold a specific responsibility for ensuring evidence-based practices.

Our study demonstrates that a KT capacity program tailored to identified needs, can strengthen the PDN role in NHs, to work more systematically with implementation. Specifically, the knowledge-to-action framework helped PDNs broaden their understanding of KT and fulfil their role in the process of involving their institution in a tailored implementation of NEWS2. Still, KT capacity in isolation has less relevance if uncoupled from a commitment permeating the organization. The perception of an organizational dedication towards common challenges and common goals in IMPAKT is likely attributable to the underlying IKT approach.

## Supplementary Information

**Additional file 1.** Outline of the educational component of the IMPAKT intervention.

**Additional file 2.** Interview guide.

## Data Availability

The data supporting the findings of this study are not publicly available as individual privacy could be compromised. The data are available from the corresponding author on reasonable request.

## Abbreviations

EBP: Evidence-based practice
IKT: Integrated knowledge translation
IMPAKT: The IMPlementation and Action of Knowledge Translation project
KT: Knowledge translation
KTA: Knowledge-To-Action framework
NEWS2: National Early Warning Score – 2nd edition
NH: Nursing home
PDN: Practice Development Nurse
RN: Registered Nurse
