# Supplementary material for: Integrated knowledge translation in nursing homes: exploring the experiences of practice development nurses
Source: BMC Health Serv Res. 2021 Nov 29;21:1283. doi: 10.1186/s12913-021-07282-7 (PMC8628377; doi:10.1186/s12913-021-07282-7)
Supplement: Supplementary file 1 — Additional file 1. Outline of the educational component of the IMPAKT intervention. [file 12913_2021_7282_MOESM1_ESM.pptx]

## Slide 1
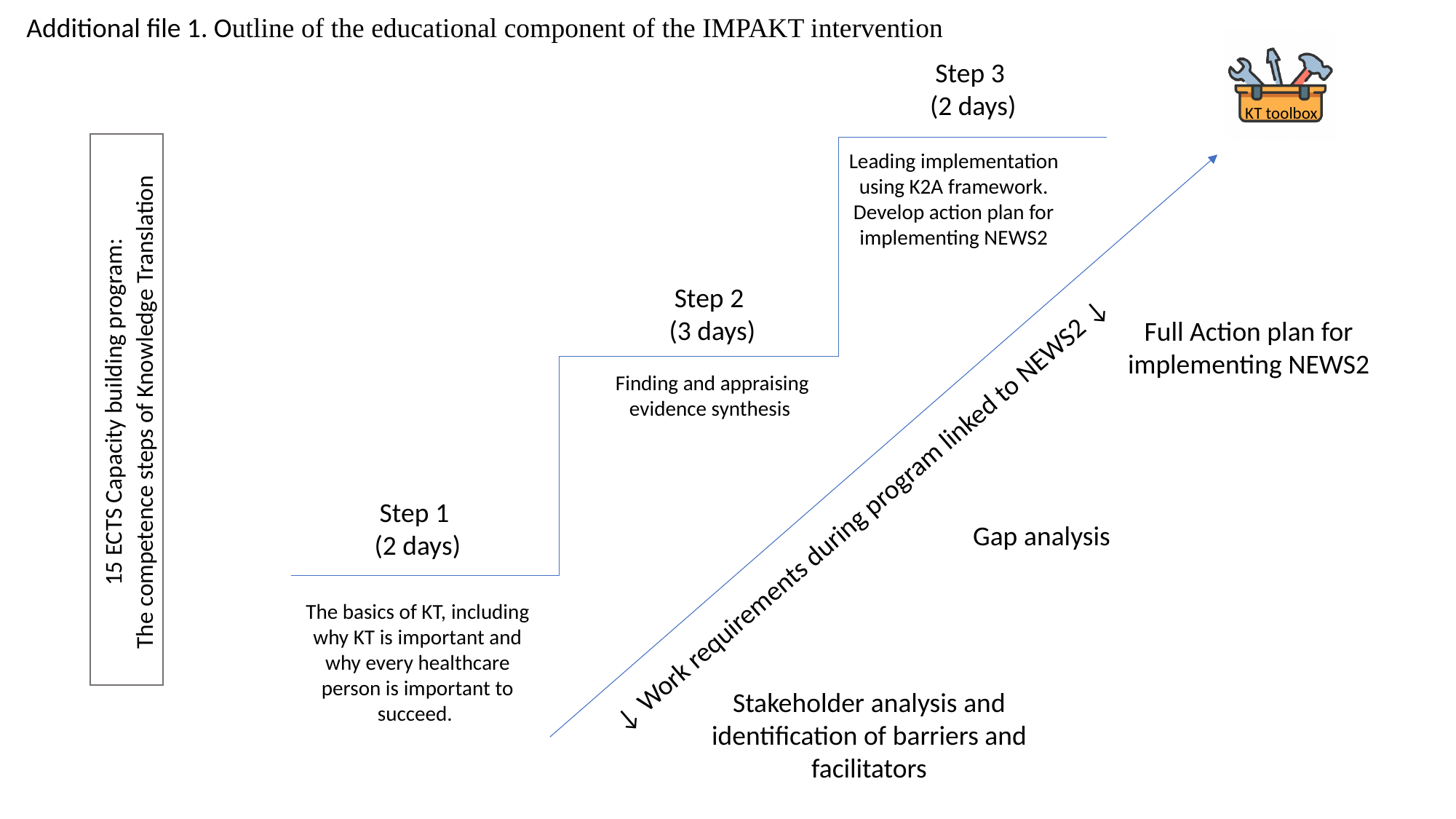

Additional file 1. Outline of the educational component of the IMPAKT intervention
Step 3
(2 days)
KT toolbox
Leading implementation using K2A framework.
Develop action plan for implementing NEWS2
Step 2
(3 days)
Full Action plan for implementing NEWS2
Finding and appraising evidence synthesis
15 ECTS Capacity building program:
The competence steps of Knowledge Translation
Step 1
(2 days)
↓ Work requirements during program linked to NEWS2 ↓
Gap analysis
The basics of KT, including why KT is important and why every healthcare person is important to succeed.
Stakeholder analysis and identification of barriers and facilitators
