# Supplementary material for: Integrated knowledge translation in nursing homes: exploring the experiences of practice development nurses
Source: BMC Health Serv Res. 2021 Nov 29;21:1283. doi: 10.1186/s12913-021-07282-7 (PMC8628377; doi:10.1186/s12913-021-07282-7)
Supplement: Supplementary file 2 — Additional file 2. Interview guide. [file 12913_2021_7282_MOESM2_ESM.docx]

| **Interview guide** | |
| --- | --- |
| 1. **The educational component** | |
| a) | Can you tell about how you experienced participating in the educational component?  - Can you point out anything especially useful?  - Can you highlight something particularly challenging? |
| b) | How do you consider the educational component has affected your understanding of presenting and introducing new knowledge in nursing homes? |
| 1. **The action plan** | |
| a) | Can you tell us about how you experienced the process of developing the action plan?  - What was most challenging?  - What was helpful? Can you elaborate on this?  - What was important to you? Can you elaborate on this?  - Do you have any thoughts of what could have been done differently? |
| b) | What would you say is the strength(s) of the action plan as a tool?  - Do you see any challenges in using it in the introduction of NEWS2 at your nursing home?  - How do you plan to face these challenges? |
| 1. **The initial implementation phase** | |
| a) | You are now starting the initial introduction of NEWS2, what thoughts do you have about this?  - How do you think this intervention can affect the nursing home practice? |
| b) | Can you tell about how you have prepared your colleagues for the NEWS2 intervention?  - How do you experience their response and motivation?  - what do you think has influenced response and motivation.  (If positive, is it possible to point out what has been done (implementation measures/motivation) that has led to this? Same if the response and motivation are negative – what could be the reason(s)?) |
| c) | What do you consider good ways to organize the work with the introduction of NEWS2 -in order for useful contributions from the nursing home staff?  - What helps to promote a positive attitude towards the introduction of NEWS2?  - Who are essential people in this context?  - How can you help create a positive attitude?  - What can reduce negative attitudes towards the introduction of NEWS2?  Who are the essential people in this context? |
| 1. **The role of the PDN** | |
| a) | Can you say something about if (and how) the participation in the educational component and the introduction of NEWS2 affects your perception of your role as a PDN? |
| b) | What thoughts do you have about your own competence and skills related to implementing NEWS2?  - Is there anything that could have contributed to you feeling more prepared? |
| c) | What will be your role in the introduction of NEWS2?  - What opportunity do you have to work with this?  - What is important to you in this?  - How do you plan to prioritize time for this?  - What does it take for you to succeed in your role as a PDN? |
| 1. **Cooperation** | |
| a) | How is the management team involved in the work with the introduction of NEWS2 in your nursing home?  - What significance has this had?  - What kind of support is important to you? |
| 1. **Closing** | |
| a) | Is the anything else you want to add? |
